# Cell adhesion and sorting in embryoid bodies derived from N- or E-cadherin deficient murine embryonic stem cells

**DOI:** 10.1242/bio.20146254

**Published:** 2014-01-03

**Authors:** Robert Moore, Wensi Tao, Yue Meng, Elizabeth R. Smith, Xiang-Xi Xu

**Affiliations:** Sylvester Comprehensive Cancer Center and Department of Cell Biology, University of Miami School of Medicine, Miami, FL 33136, USA

**Keywords:** Differential adhesive affinity hypothesis, Cell adhesion, Cell segregation, Cell sorting, Epithelial polarity, Embryos, Germinal layers, Primitive endoderm, Morphogenesis, Self-assembly

## Abstract

The primitive endoderm epithelial structure in mouse blastocysts forms following cell differentiation and subsequent sorting, and this two-step process can be reproduced in vitro using an embryoid body model. We found that in the chimeric embryoid bodies consisting of paired wildtype and E-cadherin null ES cells, the wildtype sorted to the center and were enveloped by the less adhesive E-cadherin null cells, in accord with Steinberg's hypothesis. However, wildtype and N-cadherin null ES cells intermixed and did not segregate, a situation that may be explained by Albert Harris' modified principle, which incorporates the unique properties of living cells. Furthermore, in chimeric embryoid bodies composed of N-cadherin and E-cadherin null ES cells, the two weakly interacting cell types segregated but did not envelop one another. Lastly, the most consistent and striking observation was that differentiated cells sorted to the surface and formed an enveloping layer, regardless of the relative cell adhesive affinity of any cell combination, supporting the hypothesis that the ability of the differentiated cells to establish apical polarity is the determining factor in surface sorting and positioning.

## Introduction

The groundbreaking work of Townes and Holtfreter in 1955 demystified the patterning of embryonic cells in the spontaneous morphogenesis of germ layers, and most importantly, paved the way to a new era in the study of embryology (Townes and Holtfreter, 1955; Steinberg and Gilbert, 2004). They dissociated cells from amphibian neurulae and noticed that in heterotypic mixtures, the cells were able to reorganize in a tissue specific manner (Townes and Holtfreter, 1955; Steinberg and Gilbert, 2004). The classic experiments demonstrated that dissociated embryonic cells can sort and reconstruct embryonic germinal layers upon re-aggregation, first indicating that the embryonic patterning is a result of cell spontaneous assembly and follows physical laws governing cell interactions (Steinberg and Gilbert, 2004).

The sorting behavior explains the remarkable cell dynamics of cells during in the patterning of early embryos, such as the formation of the primitive endoderm in peri-implanting mammalian blastocysts. Recently, a model was proposed that in the developing inner cell mass (ICM) of the blastocysts, primitive endoderm differentiation occurs randomly, but then the differentiated primitive endoderm cells sort to the surface to form the primitive endoderm epithelial layer (Chazaud et al., 2006). The two-step process of cell differentiation and sorting can also be recapitulated in vitro using embryoid bodies formed by aggregation of embryonic stem (ES) cells (Rula et al., 2007). Embryoid bodies mimic the early steps of spontaneous cell differentiation and morphogenesis of the early embryos and are tractable models to study these processes (Coucouvanis and Martin, 1995; Capo-Chichi et al., 2005). Such cell aggregates are popular models to explore the principles and mechanisms governing the cell sorting and positioning behaviors by developmental biologists and by mathematic modeling analysis (Armstrong et al., 2006; Brodland and Chen, 2000; Peifer, 1998; Tepass et al., 2002; Foty and Steinberg, 2005; Gumbiner, 1996; Gumbiner, 2005). The best known and comprehensive theory to explain the spontaneous sorting and patterning of the embryonic layers is the differential adhesive affinity hypothesis proposed by Malcolm Steinberg (Steinberg, 1962; Steinberg, 1963). This hypothesis postulates that less adhesive cells sort to the periphery and envelop highly adhesive cells in the center. Such an arrangement achieves lowest free energy (Steinberg, 1963). The mathematically elegant “differential adhesive affinity hypothesis” explains satisfactorily many biological phenomena (Steinberg, 2007). Additionally, Albert K. Harris provided a critique of the Steinberg hypothesis, pointing out properties of living cells may cause deviations from the rule of differential adhesive affinity in some circumstances (Harris, 1976).

Using E-cadherin null ES cells as the less adhesive component compared to wildtype, we previously demonstrated that indeed the sorting pattern is consistent with that predicted by Steinberg's hypothesis (Moore et al., 2009). However, when E-cadherin null ES cells were mixed with primitive endoderm cells differentiated from wildtype cells, adhesive affinity no longer dictates sorting pattern (Moore et al., 2009). Thus, the differential adhesive affinity hypothesis may explain sorting of undifferentiated ES cells, but it cannot explain the patterning of embryonic germinal layers. Rather, we proposed that the ability to establish an apical polarity underlies the surface positioning of primitive endoderm cells (Yang et al., 2007; Moore et al., 2009).

In early mouse embryos, E-cadherin and N-cadherin are the principle cell–cell adhesion molecules (Moore et al., 1999). Both the specificity and affinity of cell adhesion molecules may affect cell sorting (Larue et al., 1996; Friedlander et al., 1989; Katsamba et al., 2009). E-cadherin is required for compaction in morulae (Takeichi, 1991; Larue et al., 1994; Riethmacher et al., 1995). N-cadherin deletion leads to defective morphogenesis of embryonic structures (Radice et al., 1997), and N-cadherin-deficient cells are segregated in chimeric embryos (Kostetskii et al., 2001).

To explore further the rules for cell sorting in early embryos, we tested the sorting of ES cells with deficiency in cell adhesion molecules, E-cadherin and N-cadherin, in embryoid body models. In the experiments, we used three different mouse embryonic stem cell lines, GFP-labeled wildtype (CFG37), E-cadherin knockout (9J), and N-cadherin knockout (N95) to examine homotypic and heterotypic cell adhesion and how differentiation influences adhesion. Our experiments produced some unexpected results, and the study may enrich and provide further understanding on the mechanisms of cell sorting and patterning.

## Materials and Methods

### Mutant and wildtype ES cells: labeling, propagation, and differentiation

The murine embryonic stem cell lines used in this study include: RW4 (wildtype), CFG37 (wildtype, express the βACT-GFP transgene) (Moore et al., 2009; Okabe et al., 1997), Ncad19 (N-cadherin (+/−)), Ncad95 (N-cadherin (−/−)) (Moore et al., 1999), and 9J (E-Cadherin (−/−)) (Larue et al., 1996). The cells were normally maintained on feeder layers of irradiated murine embryonic fibroblasts in ES cell medium supplemented with 1,000 units/ml of recombinant LIF (ESGRO, Chemicon International), at 37°C and 5% CO_2_.

The unlabeled cells were marked by incubating with CellVue Claret reagent (Molecular Targeting Technologies, Inc.) according to the manufacturer's instructions. ES cells were differentiated into endoderm by exposure to 1 µM all trans retinoic acid for 4–7 days as monolayers cultured in gelatin-coated tissue culture dishes. Typically, 80 to 90% of the cells are differentiated as indicated by strong GATA4 or Dab2 expression detected by immunofluorescence microscopy.

### Cell aggregation assay

The rate of cell adhesion as an indication of cell adhesive affinity was measured using a Coulter Counter. GFP labeled wildtype mouse ES cell (CFG37), E-cadherin null ES cell (9J) and N-cadherin null ES cells (N95) were dissociated using trypsin/EDTA and placed in a 50 ml Falcon tube at a density of 2×10^6^ cells/ml, along with 10 ml ES cell medium buffered with 10 mM HEPES, pH 7.4. During the assay, the cells were incubated at 37°C in a gyratory shaker shuddering at 150 rpm speed. Readings for particle number in triplicate samples were taken every hour up to 3 hours with a Z1 Beckman Coulter Particle Counter set at 8 µm threshold particle size. The reduction in the particle number reading at each time point indicates the rate cell aggregation.

### Chimeric embryoid body formation

Embryoid bodies were formed from 6×10^6^ mono-dispersed ES cells, pre-differentiated or pluripotent, in a bacterial petri dish with 10 ml of ES medium, and allowed to coalesce in suspension for 1–3 days. The conditions and cell density were tested to ensure that the spheroids produced had sizes relatively even and similar to the cross section of an actual E5.5 embryo (about 100 to 200 µm in diameter). Heterotypic spheroids were made by inoculating equal numbers of two ES cell types, where one population was fluorescence-labeled and was either undifferentiated or prior differentiated. For short term (2 days) sorting experiments using only undifferentiated ES cells, LIF (1,000 units/ml) was included in the medium to reduce spontaneous differentiation. Medium without LIF was used for experiments using differentiated ES cells or in experiments intended to observe spontaneous differentiation in the embryoid bodies.

### Immunohistochemistry and immunofluorescence of spheroids/embryoid bodies

Spheroids were fixed with neutral buffered formalin, paraffin embedded and sectioned and placed on glass slides. Slides were deparaffinized in xylene, hydrated in graded ethanol series, washed in water, and boiled in antigen retrieval solution (10 mM sodium citrate, pH 6.0). Following incubation with primary antibodies, corresponding species-specific secondary antibodies were applied. For immunohistochemistry, the horseradish peroxidase (HRP) conjugated secondary antibody (Vector Laboratories) was detected by a DAB peroxidase substrate kit (Vector Laboratories) followed by a hematoxylin counterstain. For immunofluorescence, multiple secondary antibodies conjugated with the appropriate Alexa fluorochrome were used for simultaneous imaging of multiple antigens. DAPI (4′-6-diamidino-2-phenylindole) solution was used as a generic nuclear counterstain and applied at terminal stages of the procedure.

Primary antibodies used include: anti-E-cadherin (BD Transduction Labs no. 610181), anti-Dab2 (BD Transduction Labs no. 610465), anti-N-cadherin (BD Transduction Labs no. 610920), anti-beta-actin (Sigma no. A5441), anti-GATA4 (Santa Cruz Biotechnology, sc-1237), anti-Oct3/4 (Santa Cruz Biotechnology, sc-5279), and anti-megalin (Santa Cruz Biotechnology, sc-16478). SuperSignal West Extended Duration Substrate (PIERCE) was used for chemoluminescence detection of proteins on Western blots.

Conventional wide-field microscopy was performed with an inverted Zeiss AxioObserver Z1 operated by Axio Vision 4.8 software and a Plan-Apochromat ×63 (oil immersion, N/A 1.4) or A-Plan ×10 (N/A 0.25) objective. Images were acquired digitally with a monochrome Zeiss AxioCam MRm CCD camera. Confocal imaging was performed with a Zeiss LSM510/uv Axiovert 200M inverted, laser scanning confocal microscope operated by Zeiss LSM software. For live imaging, embryoid bodies were resuspended in medium buffered with 10 mM HEPES, pH 7.4, and imaged in a glass bottom microwell dish (MatTek Corporation, MA, USA) with the Plan-Neofluar ×25 lens (water immersion, N/A 0.8). Dead cells were imaged by the inclusion of the propidium iodide fluorophore in the medium.

## Results

### Cell adhesive affinity of wildtype and cadherin mutant ES cells

In early mammalian embryos following implantation of the blastocysts, the inner cell mass expands, and induction of GATA6 determines differentiation into primitive endoderm (Cai et al., 2008), which subsequently forms visceral and parietal endoderm. Within the inner cell mass, cells actively sort and position to form a primitive endoderm layer covering the surface (Rossant et al., 2003; Chazaud et al., 2006; Plusa et al., 2008; Meilhac et al., 2009; Morris et al., 2010; Frankenberg et al., 2011), and cell adhesion molecules including E-cadherin and N-cadherin mediate the cell–cell interactions (Gumbiner, 1996; Gumbiner, 2005; Moore et al., 2009). We have collected available wildtype and mutant ES cells with specific mutation of these cell adhesion genes, GFP labeled wildtype (CFG37), E-cadherin knockout (9J) (Larue et al., 1996), and N-cadherin knockout (N95) (Moore et al., 1999), to examine homotypic and heterotypic cell adhesion and how differentiation influences cell sorting.

**Fig. 1.**
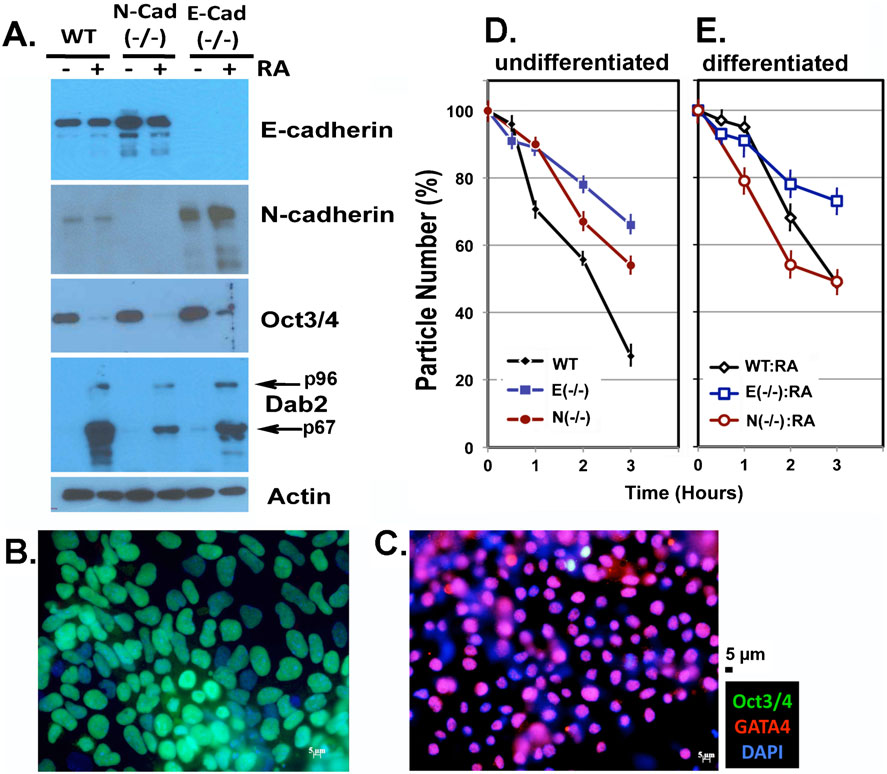
Cell adhesion molecule expression and the aggregation of wildtype and mutant ES cells. (A) Wildtype (WT), E-cadherin null (9J), and N-cadherin null (Ncad95) ES cells, with or without differentiation by retinoic acid, were analyzed by Western blot for the proteins levels of E-cadherin and N-cadherin. Oct3/4 and Dab2 levels are indicators of ES cell pluripotency or endoderm differentiation, respectively. The intensities of the signal in the Western blots were quantified using Image J program. (B,D) ES cells in monolayer cultures were analyzed by immunofluorescence microscopy by staining for Oct3/4, GATA4, and DAPI prior to (B), or following differentiation with 1 µM retinoic acid for 5 days (C). The representative individual images acquired were overlaid to produce the composed figures shown. (D) Rate of aggregation of the ES cells was determined as a measure of cell adhesive affinity. Cells were first mono-dispersed, washed with cold PBS, and then allowed to aggregate at 37°C. The aggregation of undifferentiated cells was measured using a Coulter Counter and the reduction of particle number is presented. (E) Wildtype, E-cadherin null (9J), and N-cadherin null (Ncad95) ES cells were first differentiated by treatment with retinoic acid for 4 days. The aggregation of the differentiated cells was measured using a Coulter Counter and the reduction of particle number is presented. Coulter Counter reading of particle numbers were performed using triplicate samples and the average and standard error are reported. Scale bars: 5 µm.

First, the expression of E-cadherin and N-cadherin was determined in wildtype, 9J (E-cadherin null), N19 (N-cadherin heterozygous), and N95 (N-cadherin null) ES cells, with or without prior differentiation by retinoic acid treatment (Fig. 1A). E-cadherin expression was increased (2.1-fold) in N-cadherin-null ES cells (Fig. 1A). A more pronounced increase in N-cadherin was detected in E-cadherin knockout ES cells (6.2-fold), and the increase was further magnified upon RA-induced differentiation (to 7.9-fold). Both E-cadherin or N-cadherin null ES cells were differentiated upon treatment with retinoic acid (RA), as indicated by the loss of Oct3/4 and the induction of Dab2 (Fig. 1A). We also used immunofluorescence microscopy to determine the degree of differentiation. Prior to treatment with retinoic acid, the ES cells were largely Oct3/4-positive, although with variable intensity among individual cells (Fig. 1B). The undifferentiated cells showed few (undetectable in this image) GATA4-positive cells (Fig. 1B). Retinoic acid treatment of the ES cells on monolayer cultures led to the loss of Oct3/4 in the majority of the cells, which were mostly (80–90%) GATA4-positive (Fig. 1C). The changes in nuclear morphology of ES cells upon differentiation were likely caused by expression of nuclear envelope proteins (Smith et al., 2011).

We then characterized and compared the mutant ES cells for cell adhesive affinity and ability to aggregate, using a Coulter Counter to measure the reduction of particle number and thus the rate of cell aggregation. As shown in Fig. 1D, deletion of either E-cadherin or N-cadherin reduced cell aggregation, although the deletion of E-cadherin had a greater reduction in the speed of aggregate formation than the deletion of N-cadherin. We also determined the aggregation of differentiated cells. By immunofluorescence microscopy analysis using markers such as GATA4 and Oct3/4, we determined that 80–90% of the ES cell population had differentiated into extraembryonic endoderm-like cells after treatment with retinoic acid (Fig. 1B,C). Differentiation reduced cell aggregation of wildtype ES cells, but slightly increased that of N-cadherin null cells (Fig. 1E). Although we determined and concluded that the wildtype ES cells had higher adhesive affinity than N-cadherin null cells, and the E-cadherin null ES cells had the lowest adhesive affinity (Fig. 1D), the differences in cell–cell adhesion rate appeared not to correlate with expression levels of E-cadherin and N-cadherin (Fig. 1A). For example, N-cadherin null ES cells (total cadherin = 2.1E+0N) expressed 2.1-fold of E-cadherin compared to wildtype (total cadherin = 1E+1N), yet showed a slight reduced cell adhesion; and E-cadherin null ES cells (total cadherin = 0E+6.2N) had 6.2-fold N-cadherin to compensate for the loss of E-cadherin, but exhibited lowest adhesive affinity. It appears that the apparent levels of E-cadherin and N-cadherin cannot predict adhesive affinity of the cells when one of the cadherin is deleted.

### Sorting between wildtype and N-cadherin or E-cadherin null ES cells in chimeric embryoid bodies

**Fig. 2.**
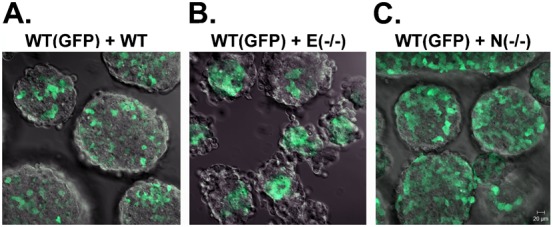
Sorting in chimeric embryoid bodies of wildtype with E- or N-cadherin null ES cells. Wildtype, E-cadherin null (9J), and N-cadherin null (Ncad95) ES cells were mixed with CFG37 ES cells, a wildtype line that stably express the βACT-GFP transgene, to form chimeric embryoid bodies. The two cell types were first dispersed into single cells with EDTA and trypsin, and 3×10^6^ of each cell type were plated and maintained in suspension culture in 10 ml medium on bacteriological plastic plates for 2 days. The aggregates formed were harvested and placed in a microwell dish for image analysis. Confocal images of GFP were acquired by vertical sectioning and a representative image near the middle section is shown for each type of chimeric embryoid bodies. Heterotypic Spheroids: (A) CFG37 + RW4; (B) E-cadherin null + CFP37; and (C) N-cadherin null + CFP37. Scale bar: 20 µm.

We used GFP-labeled wildtype ES cells to mark one population, and an equal numbers of wildtype or mutant ES cells were dispersed as single cells, intermixed evenly, and allowed to aggregate into spheroids (embryoid bodies). We started with a control experiment by mixing two wildtype ES cells, unlabeled RW4 wildtype with GFP-labeled CFG37. When equal amount of mono-dispersed ES cells were mixed and incubated in suspension for 2 days, the chimeric cell aggregates showed a checkerboard pattern of intercalated cells (Fig. 2A).

When intermixed as dispersed cells, E-cadherin null ES cells segregated and sorted to the surface layer of the chimeric embryoid bodies leaving the GFP-labeled wildtype cells at the core (Fig. 2B), consistent with that previously shown (Moore et al., 2009). The less adhesive E-cadherin null ES cells localized on the outer layer, which enveloped the highly adhesive wildtype ES cells found interior, a sorting pattern that agreed with the prediction based on Steinberg's differential adhesive affinity hypothesis.

However, surprisingly, no obvious cell sorting occurred in wildtype and N-cadherin null chimeric embryoid bodies (Fig. 2C). In the spheroids, the two different cell types intermingled and no segregated pattern was apparent (Fig. 2C). We repeated the experiments three times where we varied the time courses and ratio of the two cell types, but in each case no overt segregation was observed, even following an extensive incubation (5 days). Nevertheless, careful examination suggested that there were subtle differences in the distribution of labeled wildtype and unlabeled N-cadherin null ES cells. Comparing to the wildtype mix (Fig. 2A), the frequency of contacts between individual labeled WT cells appeared to be increased in the aggregates from the mixtures of GFP-WT and N-cadherin null ES cells (Fig. 2C). For example, about 30% of GFP-labeled cells appeared isolated without contact with other GFP-positive cells in the mixture of wildtype cells (Fig. 2A). The majority of GFP-positive cells were in close contact with at the least one GFP-positive cell in the mixture of wildtype and N-cadherin null cells, and less than 5% of GFP-positive cells appeared isolated (Fig. 2C). Although we also recognized that the variation of ratio between labeled and unlabeled cells within each aggregate may influence the observation, such subtle cell adhesion behavior would require sophisticated image tracking method for further quantization.

Thus, although differential cell adhesive affinity could be determined, no overt cell sorting was achieved when wildtype and N-cadherin null ES cells were mixed. This result was unexpected and might be explained by two possibilities as discussed below.

### Sorting between N-Cadherin and E-cadherin null ES cells in chimeric embryoid bodies

**Fig. 3.**
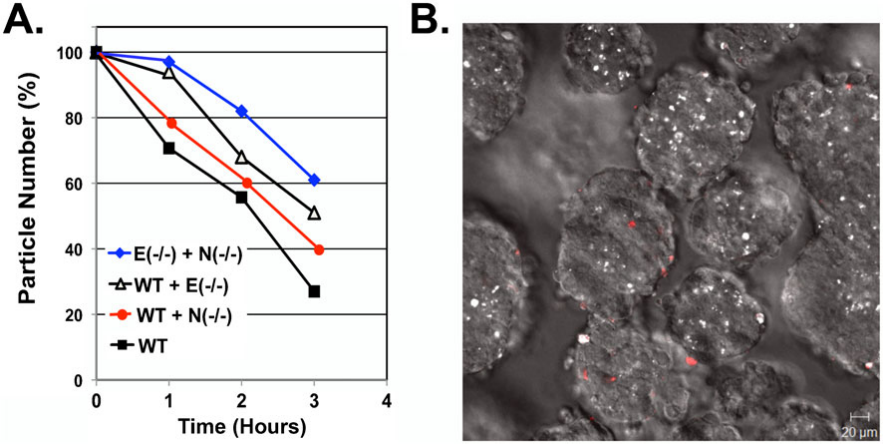
Weak interaction between E-cadherin and N-cadherin null cells. (A) Equal numbers of two different types of ES cells were allowed to aggregate at 37°C at a cell density of 2×10^6^ cells/ml. The rate of aggregation of the mixture was measured using a Coulter Counter, and the reduction of particle number is presented. The aggregation of wildtype (WT) ES cells at the same density is used for comparison. Particle number was determined in triplicate samples and the difference in the value was smaller than 5%. N(−/−): N-cadherin null; E(−/−): E-cadherin null. (B) N-cadherin null ES cells were first labeled with the CellVue Claret (CVC) procedure, and then were mixed with E-cadherin null ES cells to form chimeric embryoid bodies following maintaining in suspension culture for two days. The location of CVC-labeled cells in the embryoid bodies was visualized by confocal fluorescence microscopy (bright white spots). A confocal section near the equatorial region is shown. Propidium iodide (red) was added to mark dead cells. Scale bar: 20 µm.

We also mixed E-cadherin and N-cadherin null ES cells to determine the sorting pattern in embryoid bodies. Generally, it is thought that cells interact through homophilic cadherin ligation (Miyatani et al., 1989; Nose et al., 1988), although some reported the possible heterotypic E-cadherin and N-cadherin association (Niessen and Gumbiner, 2002; Prakasam et al., 2006; Shi et al., 2008). Nevertheless, we observed the weakest interaction between E-cadherin and N-cadherin null cells, since the cell mixtures containing the two cell types aggregated slowest (Fig. 3A). In the experiments, we monitored the aggregation of a defined number of cells consisting of equal number of each cell types. The rate would be determined by the sum of both homotypic and heterotypic binding. The rate of aggregation was determined to be: WT+WT>WT+N-cadherin (−/−)>WT+E-cadherin (−/−)>N-cadherin (−/−)+E-cadherin (−/−) (Fig. 3A). The results can be explained by a greater contribution of E-cadherin than N-cadherin to cell–cell adhesion between ES cells. Initially, we attempted to identify a sorting pattern by labeling one cell type with CellVue Claret (CVC). However, in the combination of E-cadherin and N-cadherin knockout ES cells, the pattern was not obvious because only about 30% of cells retained the CVC label following culturing (Fig. 3B). We included propidium iodide (Fig. 3B, red) to track dead cells, and determined that apoptotic cell death was not wide spread and cavitation was not initiated at the early time course of cell aggregation.

**Fig. 4.**
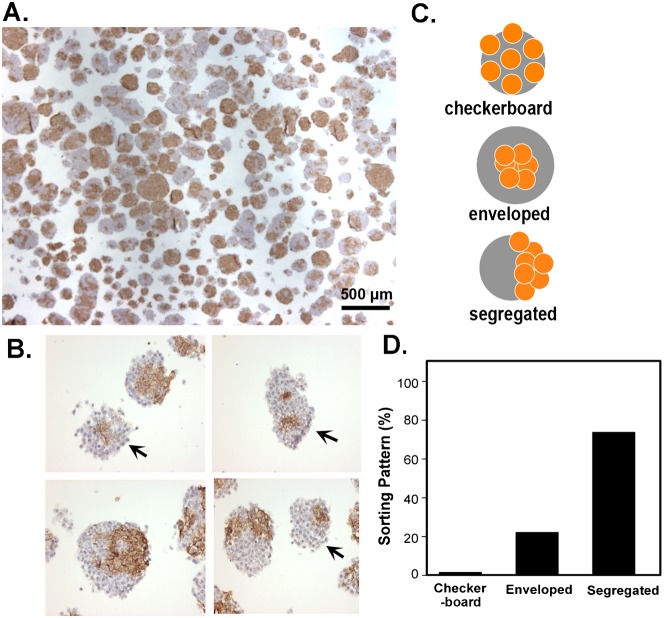
Sorting pattern and histological analysis of chimeric embryoid bodies composed of E-cadherin and N-cadherin knockout ES cells. Chimeric aggregates produced from a mixture of equal number of E-cadherin and N-cadherin null ES cells following a two-day culture were subjected to histological and immunohistochemical analysis. (A) E-cadherin immunostaining was used to distinguish E-cadherin null from N-cadherin null ES cells in the chimeric embryoid bodies. (B) Several representative examples of the chimeric embryoid bodies are shown for various sorting patterns. Aggregates indicated by an arrow are classified as enveloped, and the rest of the examples are segregated and bordering. (C) Schematic illustration of the checkerboard, enveloped, and segregated sorting patterns defined. (D) Quantification of the sorting patterns in chimeric aggregates between E-cadherin and N-cadherin null ES cells. Scale bar: 500 µm.

Nevertheless, we resorted to use E-cadherin immunostaining to distinguish the two cell types, and were able to identify several sorting and segregation patterns, as showed in the histology and the representative examples in Fig. 4A,B. The E-cadherin or N-cadherin null ES cells in the chimeric spheroids were distinguished by immunostaining of E-cadherin, since we observed that in wildtype and N-cadherin (−/−) spheroids, E-cadherin was positive in all cells. Among the sorting patterns in various spheroids (Fig. 4A), two prevalent patterns were observed: segregation and enveloping, and segregation and bordering, as shown by several examples (Fig. 4B). Only a minimal fraction (<1%) of the aggregates were composed exclusively of one cell type (uniformly E-cadherin positive or negative). To quantitate the result, we defined and classified each spheroid into either enveloping or bordering patterns (Fig. 4C), and estimated that 82% of these patterns could be categorized as segregation and bordering, 17% as enveloping, and less than 1% were not sorted (checkerboard pattern) (Fig. 4D). Thus, the two cell types appear to sort and segregate but do not envelop each other.

### Sorting between differentiated and undifferentiated ES cells in chimeric embryoid bodies

**Fig. 5.**
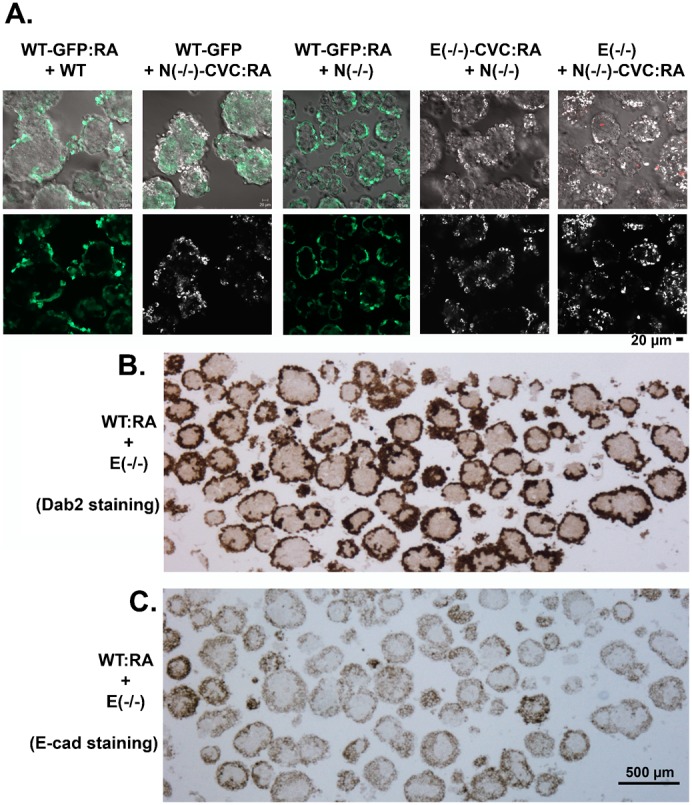
Differentiated cells sort to the outer layer, regardless of E- or N-cadherin status. Wildtype and mutant ES cells were distinguished by GFP expression or labeling with CVC. One cell type was first differentiated with retinoic acid for 4 days. Two cell types, one differentiated and one undifferentiated, were dispersed into single cells and allowed to mix and aggregate to form chimeric embryoid bodies for two days. (A) The aggregates formed were harvested and imaged by confocal sectioning to visualize both GFP and CVC labellings. A representative confocal image of GFP or CVC near the middle section is shown for each type of chimeric embryoid body in the top panels, and corresponding overlayering with DIC images is shown in the upper panels. Propidium iodide was added to visualize dead cells (red). (B,C) The chimeric embryoid bodies composed of highly adhesive wildtype ES cells that were differentiated by treatment with retinoic acid and loosely adhesive E-cadherin null cells were harvested and used for histology and immunohistochemistry. Immunostaining of Dab2 (B) and E-cadherin (C) were performed on two adjacent slides to visualize the differentiated wildtype (Dab2 and E-cadherin positive) and undifferentiated E-cadherin null (Dab2 and E-cadherin negative) ES cells. Scale bars: 20 µm (A), 500 µm (B,C).

We also examined the effect of differentiation on cell sorting. We tested chimeric embryoid bodies composed of all possible combinations of genotypes of differentiated and undifferentiated ES cells, including WT+WT:RA, WT+N(−/−):RA, WT:RA+N(−/−), E(−/−):RA+N(−/−), N(−/−):RA+E(−/−), and WT:RA+E(−/−). When GFP-labeled wildtype ES cells were differentiated and mixed with non-labeled ES cells, and allowed to sort for 2 days, without exception, differentiated cells sorted to the outer layer, regardless of the combination of E- or N-cadherin status (Fig. 5A). When one cell type was labeled with CVC, the surface sorting of differentiated cells was still apparent despite that only around 30% of the cells remained positive (Fig. 5A). In embryoid bodies in which the two constituent cell types had the most extreme difference in binding affinity, the highly adhesive wildtype ES cells when differentiated, nevertheless, enveloped the weakly adhesive E-cadherin null ES cells (Fig. 5B,C). The differentiated Dab2-positive endoderm cells occupied the outer layer (Fig. 5B), which also stained positive for E-cadherin, with the undifferentiated inner core negative for E-cadherin (Fig. 5C).

When one cell type was differentiated, the most consistent and striking observation was that differentiated cells sort to the surface and form an enveloping layer regardless the relative cell adhesive affinity of any combination in the chimeric embryoid bodies, further substantiating our previous finding (Moore et al., 2009). Thus, differentiated cells no longer follow Steinberg's differential adhesive affinity hypothesis (Steinberg, 1962; Steinberg, 1963; Steinberg, 2007).

### Establishment of apical polarity of primitive endoderm cells upon arriving on the surface

**Fig. 6.**
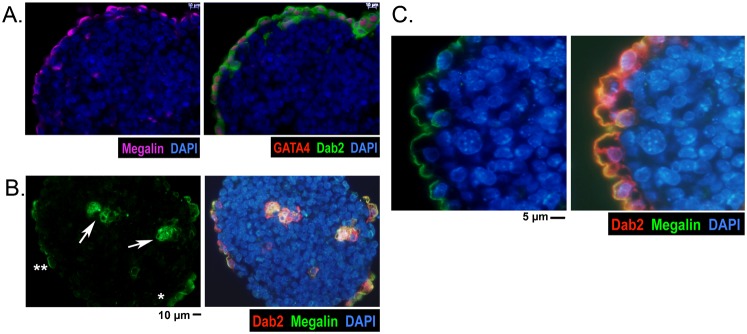
Apical polarity of primitive endoderm cells on surface of embryoid bodies. Embryoid bodies were analyzed by immunofluorescence microscopy for GATA4 (primitive endoderm marker), Dab2 (primitive endoderm marker), and megalin (primitive endoderm apical polarity marker). (A) A representative embryoid body derived from wildtype ES cells. Dab2 (cytoplasmic) and GATA4 (nuclear) mark the surface layer of primitive endoderm cells. Megalin is restricted to the apical membrane; (B) A 4-day-old embryoid body derived from E-cadherin null ES cells is shown for example. Dab2 staining marks both internal and surface primitive endoderm cells. Megalin staining indicates that primitive endoderm cells located inside the spheroid shows no polarity, and the cells located on surface display various degree of apical polarization (degree of megalin apical restriction), from less (indicated by *) to more (indicated by **) localization to apical surface. (C) A more mature (7-day-old) embryoid body derived from E-cadherin null ES cells shows strong apical polarity of megalin of the primitive endoderm cells on surface. Scale bars: 10 µm (A,B), 5 µm (C).

We speculate that the cell autonomous ability to produce an apical polarity may account for the positioning of the differentiated primitive endoderm cells on the surface of the embryoid bodies. As an example of a representative embryoid body derived from wildtype ES cells, the cell surface glycoprotein megalin is restricted to the apical membrane of the surface layer of GATA4- and Dab2-positive primitive endoderm cells (Fig. 6A). The most surprising examples are embryoid bodies derived from E-cadherin null ES cells, in which primitive endoderm cells still sort to the surface and establish apical polarity (Fig. 6B). However, when not located on the surface, megalin does not show polarized distribution when the Dab2- and GATA4-marked primitive endoderm cells reside in the interior of the spheroids (arrow, Fig. 6B). In the 4-day-old embryoid bodies, each of the surface-located primitive endoderm cells appeared to be in various stages of polarization, as shown by the degree of megalin localization to the apical position (Fig. 6B). Megalin staining appears more diffused and cytoplasmic in some surface cells (indicated by *, Fig. 6B) than other cells (indicated by **, Fig. 6B) in the same spheroid. In more mature (7-day-old) embryoid bodies, surface primitive endoderm cells consistently showed a higher degree of apical distribution of megalin despite the absence of E-cadherin (Fig. 6C). The progressive localization of megalin to the apical surface has also been described previously for primitive endoderm cells of wildtype blastocysts (Gerbe et al., 2008).

Thus, primitive endoderm cells are not polarized when located interiorly. The various degree of polarization of the primitive endoderm cells on surface suggests that polarity of the cells forms gradually when the cells arrive at the surface. The apical polarity is established as the cells are fixed on surface to form an epithelium, and we infer that polarity prevents the primitive endoderm cells moving inward and maintains surface positioning (Yang et al., 2007).

## Discussion

The mechanisms and principles on how cells sort and form pattern spontaneously have been explored and speculated since the experiments on amphibian embryonic cells by Townes and Holtfreter (Townes and Holtfreter, 1955). However, a clear explanation of embryonic cell sorting and patterning has not yet been reached and cell sorting theories are still being debated (Amack and Manning, 2012; Green, 2008). To add to the understanding of cell sorting and patterning of early embryos, in the current study we used the murine embryoid body system and mutant ES cells with a deficiency in cell adhesion molecules, either E-cadherin or N-cadherin, to analyze cell sorting and positioning. The experimental results have uncovered several new salient points on the mechanism of cell sorting and positioning.

As shown earlier in chimeric embryoid bodies (Moore et al., 2009), the less adhesive E-cadherin deficient ES cells sort to the periphery and envelop the more adhesive wildtype ES cells, a pattern predicted by Steinberg's differential adhesive affinity hypothesis. Although N-cadherin null ES cells are less adhesive than wildtype, N-cadherin null ES cells do not sort from wildtype ES cells, but the two cell types form a random, checkerboard pattern in chimeric embryoid bodies. One argument is that the reduced binding affinity of the N-cadherin null ES cells may be miniscule, and the aggregation assay does not truly indicate cell adhesive affinity because the assay measures the speed of aggregation. However, the speed of cell aggregation generally does reflect cell adhesive affinity. This result raises a question on the threshold of the difference in adhesive affinity required between two cell types before sorting can be accomplished. A possible explanation was suggested by Harris (Harris, 1976), that living cells are not a closed system regarding entropy as considered in Steinberg's theory. Indeed, living cells produce energy from metabolism and have active locomotion. The metabolic energy generated might prevail over the reduction of free energy when an optimal cell sorting pattern is achieved. Thus, presumably cell locomotion driven by metabolic energy can overcome the energy disparity in the choice of cell adhesion between wildtype and N-cadherin null. However, the energy difference in heterotypic versus homotypic binding between wildtype and E-cadherin null ES cells is higher than cell metabolism can generate, and segregation between wildtype and E-cadherin null cells occurs. Thus, whether two different types of cells are able to sort or not will depend on both the energy the living cells are able to produce from metabolism and the difference in adhesive affinity.

Additionally, N-cadherin deletion, although it affects embryonic development (Radice et al., 1997) and the N-cadherin-deficient cells segregate in chimeric embryos (Kostetskii et al., 2001), does not show evidence of abnormality at the blastocyst or peri-implantation stages. The lack of an in vivo cell sorting phenotype was also reported in a study of Xenopus cells and embryos (Ninomiya et al., 2012).

The patterns of cell sorting in the chimeric embryoid bodies, either enveloping or bordering/segregating, can be explained by the physical principle that the arrangements achieve the lowest energy by satisfying the strongest cell–cell bonding. In the case of wildtype (WT) plus E-cadherin null (E(−/−)) ES cells, the strength of the cell–cell bonding is: WT+WT>WT+E(−/−)>E(−/−)+E(−/−). Here, both E-cadherin and N-cadherin from WT cells can interact with N-cadherin of E(−/−) cells, and hence WT+E(−/−) is stronger than E(−/−)+E(−/−) interaction. The arrangement of E(−/−) enveloping WT cells will ensure the maximal contact between WT to WT, followed by WT+E(−/−), and least E(−/−)+E(−/−). In chimeric embryoid bodies composed of E(−/−) and N(−/−) cells, the bonding strength is: N(−/−)+N(−/−)>E(−/−)+E(−/−)>N(−/−)+E(−/−). The segregation/bordering arrangement will achieve the maximal interaction between homophilic N(−/−), and then homophilic E(−/−), and minimal contact between N(−/−) and E(−/−) cells.

The current study using chimeric embryoid bodies composed of various genotypes of differentiated plus undifferentiated ES cells reinforces the idea that the ability to form apical polarity is the basis for sorting and surface positioning of endoderm cells in the embryos (Moore et al., 2009). In various combinations of differentiated and undifferentiated, wildtype and mutant ES cells, differentiated ES cells consistently sort and position on surface regardless of cell adhesive affinity. Based on the results, we can postulate the processes in the sorting and surface positioning of primitive endoderm cells as follows. In the inner cell mass or embryoid bodies with a size similar to an early embryo, the intercalated embryonic cells are in active locomotion against each other (Chazaud et al., 2006; Rula et al., 2007). Differentiated primitive endoderm cells are not polarized when located in the interior. Once the primitive endoderm cells reach the surface through random movement, an apical polarity is actively established through endocytic transport. The apical domain is enriched with bulky glycoproteins and devoid of cell adhesion molecules, and presents a non-adhesive surface (Yang et al., 2007). Hence, once polarity is established, the primitive endoderm cells are fixed at the surface.

One possibility is that differential adhesive affinity may actually contribute towards the positioning of primitive endoderm cells in the outer layer. However, this is not essential since in the chimeric embryoid bodies composed of differentiated wildtype and undifferentiated E-cadherin null ES cells, the wildtype primitive endoderm cells with a high adhesive affinity are capable of forming an outer epithelial endoderm layer. Perhaps, the embryonic phenotype of *dab2* mutant mice provides the most revealing clues on embryonic cell sorting and tissue formation (Yang et al., 2002; Yang et al., 2007). In both Dab2-deficient embryos and embryoid bodies, differentiated endoderm cells are not efficiently positioned at the surface, but intermix with epiblast cells. Dab2 is an endocytic adaptor and mediates directional vesicular transport and establishes polarity, and hence positions endoderm cells at the surface (Yang et al., 2007).

In summary, we have determined the sorting patterns of differentiated and undifferentiated, wildtype, E-cadherin or N-cadherin deficient ES cells using chimeric embryoid bodies as a model. Confirming an earlier study (Moore et al., 2009), the current results consistently support that polarity plays a dominant role over binding affinity alone, thus dictating surface positioning. We conclude that the sorting and positioning of primitive endoderm as the outer layer in early mammalian embryos are driven by the ability of the primitive endoderm cells to establish an apical polarity. Sorting between undifferentiated cells follows Steinberg's differential adhesive affinity hypothesis, such as the sorting and enveloping of E-cadherin null and wildtype ES cells. However, when the two cell types have minimal adhesive affinity towards each other, such as in the case of E-cadherin null to N-cadherin null ES cells, a segregated rather than enveloped pattern is preferred. Cadherin-mediated adhesion occurs primarily in a homophilic manner, and interaction between E-cadherin null and N-cadherin null cells is very weak. The finding that N-cadherin null ES cells are unable to sort from wildtype ES cells indicates that sufficient difference, or a threshold, in adhesive affinity is required for segregation and sorting.

Understanding spontaneous cell sorting and morphogenesis is important in tissue engineering and regenerative medicine. The current study accentuates that the seemingly simple problem of cell sorting may still be actually complex and interesting, and a mystery to be solved by the biologists, physicists and mathematicians alike.
